# MRGBP: A New Factor for Diagnosis and Prediction of Head and Neck Squamous Cell Carcinoma

**DOI:** 10.1155/2022/7281120

**Published:** 2022-07-25

**Authors:** Cheng Zhao, Chuang Wei, Xiatian Chen, Peifeng Li

**Affiliations:** ^1^Institute for Translational Medicine, Qingdao University, Qingdao 266000, China; ^2^School of Basic Medicine, Qingdao University, Qingdao 266000, China

## Abstract

MRG-binding protein (MRGBP) is a transcription factor widely involved in physiological and pathological processes. Many studies have discussed the relationship between the expression level of MRGBP and the prognosis of various malignant tumours. However, the role and clinicopathological significance of MRGBP in head and neck squamous cell carcinoma (HNSC) are unclear. In this study, the Wilcoxon signed-rank test and logistic regression were used to analyze the relationship between clinical characteristics and MRGBP expression in HNSC. The Kaplan-Meier plotter analysis and Cox regression analysis were established to evaluate the effect of MRGBP on prognosis, and the receiver operating characteristic (ROC) curve and nomogram was constructed. Gene set enrichment analysis (GSEA) and single-sample gene set enrichment analysis (ssGSEA) were used to analyze the correlation between MRGBP and immune infiltration. The results showed that the expression of MRGBP in HNSC tissues was significantly higher than that in normal tissues. The KM plotter analysis showed that the OS of HNSC patients was shorter. The multivariate Cox analysis further confirmed that increased expression of MRGBP was an independent risk factor for OS in HNSC patients. In addition, ROC analysis confirmed its diagnostic value and constructed prognostic nomograms, including age, T, M, N classification, pathological stage, and MRGBP. GSEA showed that MRGBP was associated with high expression of GPCR ligand binding, interleukin receptor binding, and neutrophil degranulation, and ssGSEA showed that MRGBP was associated with T cells and mast cells. In conclusion, MRGBP can serve as an independent prognostic biomarker related to immune invasion of head and neck squamous cell carcinoma.

## 1. Introduction

In recent years, the number of patients with head and neck squamous cell carcinoma (HNSC) has been on the rise. For example, thyroid tumours, as a type of HNSC, account for up to 4.9% of female tumours (https://www.uicc.org/news/globocan-2020-new-global-cancer-data). The mechanism of HNSC development is complex, involving changes in multiple genes and multiple signaling pathways [1, 2]. In this process, viral, environmental, and genetic factors affect the regulation and abnormal expression of tumour-related genes [3, 4]. Due to the hidden physiological location of HNSC, early diagnosis is complex, and lymph node metastasis and distant metastasis are prone to occur. Therefore, screening biomarkers are helpful to the diagnosis and pathological indicators of tumour development. MORF4-related gene-binding protein(MRGBP), a protein encoded by FLJ10914 ORF, was first reported in 2003 and is associated with the structure and function of the mammalian TRRAP/TIP60 histone acetyltransferase complex [5].

More and more researchers have begun to explore the relationship between MRGBP and tumours in recent years. Studies have shown that MRG-binding protein (MRGBP) is upregulated in most colorectal tumours [6]. However, the potential role of MRGBP in the development or metastasis of HNSC remains unclear.

In the tumour microenvironment, immune and stromal cells are two main nontumour components. The degree of tumour immune invasion and mesenchymal cells is essential for tumour diagnosis and prognosis evaluation [7]. This study comprehensively analyzed the expression of MRGBP in the cancer database and its relationship with the prognosis of cancer patients. We then used clinical specimens in the TCGA database for validation of gene expression. In addition, we investigated the relationship between MRGBP and tumour-filtering immune cells in HNSC using tumour immune evaluation resources. This study comprehensively analyzed the expression of MRGBP in the cancer database and its relationship with the prognosis of HNSC patients.

## 2. Materials and Methods

### 2.1. Patient Data Set

All raw HNSC data, including transcriptome RNA sequence data and corresponding clinical information, were downloaded from the TCGA database (https://tcga-data.nci.nih.gov/tcga/) [8]. Unless otherwise stated, all analyses were performed using RNAseq data from TCGA database HNSC in level 3 HTSEQ-FPKM format, 502 HNSC tissues and 44 paracancer tissues.

### 2.2. Gene Set Enrichment Analysis (GSEA)

GSEA, a method to reveal genomic expression data through basic knowledge, is performed to identify high-risk populations' potential biological processes and pathways in a “cluster analysis” R package [9, 10]. KEGG (Kyoto Encyclopedia of Genes and Genomes) and GO (Gene Ontology) data were downloaded from the Molecular Signature Database (MSigDB). Gene sets with |NES|>1, NOM *P* < 0.05, and FDR < 0.05 were considered statistically significant.

### 2.3. Immune Cell Infiltration of ssGSEA

Single-sample gene set enrichment analysis (GSA) in the “GSVA” R package [11] was used to analyze the immune infiltration of HNSCS, and the infiltration levels of 24 resistant cell types [12] were quantitatively analyzed according to the gene expression profile. The Spearman correlation was used to analyze the relationship between immune cell infiltration and MRGBP expression. The Wilcoxon rank-sum test was used to analyze the correlation between the degree of immune cell infiltration and MRGBP expression.

### 2.4. Statistical Analysis

The expression of MRGBP in the tissues of HNSC patients was analyzed by box plot with normal tissues of GTEx samples as control. The median method expressed by MRGBP was selected as the cut-off value. Then Wilcoxon sign rank and logistic regression were used to analyze the relationship between clinical features and MRGBP expression in HNSC. The overall survival (OS) of the high and low expression groups was analyzed by the Kaplan-Meier plotter (http://kmplot.com/ analysis). The diagnostic value of MRGBP expression was evaluated by the receiver operating characteristic (ROC) curve, and the area under the ROC curve was used as the diagnostic value. The univariate and multivariate Cox analyses were performed on TCGA-HNSC data sets to screen for potential prognostic factors. Subsequently, the independent prognostic factors of MRGBP expression were verified by the multivariate Cox analysis, and a nomogram was constructed to predict the OS at 1, 3, and 5 years in HNSC patients. The MRGBP expression level in patients with HNSC was further verified in the TIMER database (https://cistrome.shinyapps.io/timer/) [13].

## 3. Results

### 3.1. The Relationship between High Expression of MRGBP and Clinicopathological Features in HNSC Patients

**Figure 1:**
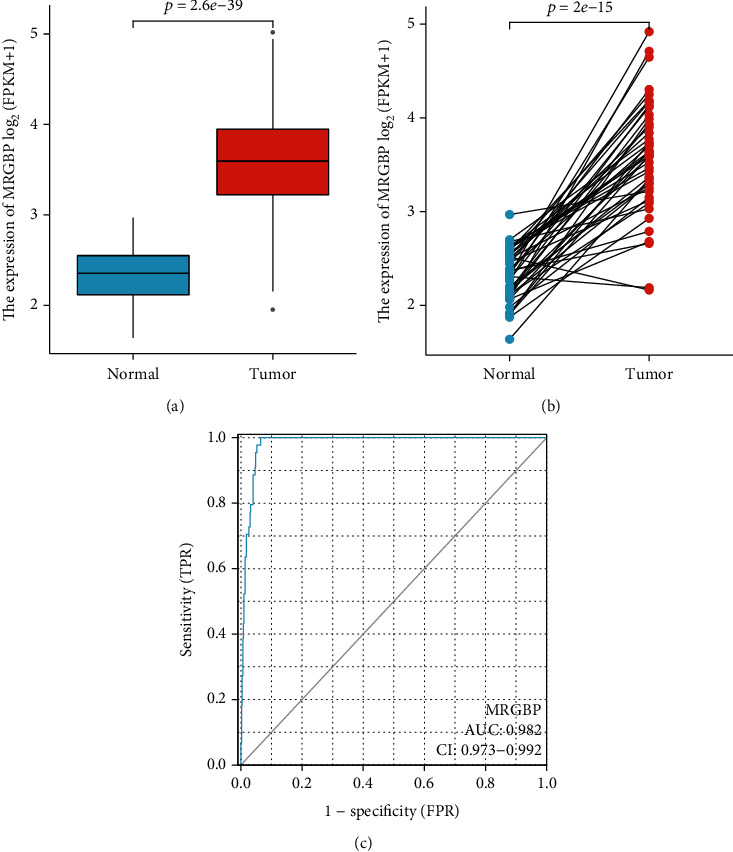
The expression of MRGBP in HNSC patients was analyzed by TCGA database. Wilcoxon signed rank-sum test was used to analyze the differential expression of MRGBP in HNSC tissues and adjacent HNSC tissues. (b) The diverse expression of MRGBP in 43 HNSC pieces and matched adjacent samples. (c) ROC curve for MRGBP in standard models of GTEx combined adjacent HNSC tissues and HNSC samples.

Comparing the expression of MRGBP in 502 HNSC tissues and 44 paracancer tissues, it was found that the expression level of MRGBP in HNSC tissues was significantly higher than that in paracancer tissues (*P* = 2.6E − 39) (Figure 1(a)). Meanwhile, MRGBP expression increased in tumour tissues of 43 HNSC samples after reservation paired sample screening (*P* = 2E − 15) (Figure 1(b)). In addition, the effectiveness of MRGBP expression in normal GTEx combined with adjacent HNSC tissues and HNSC samples was analyzed using receiver operating characteristic (ROC) curves. The area under the MRGBP curve is 0.982, which has a high diagnostic value (Figure 1(c)).

### 3.2. Relationship between MRGBP Gene Expression and Clinical Features

**Figure 2:**
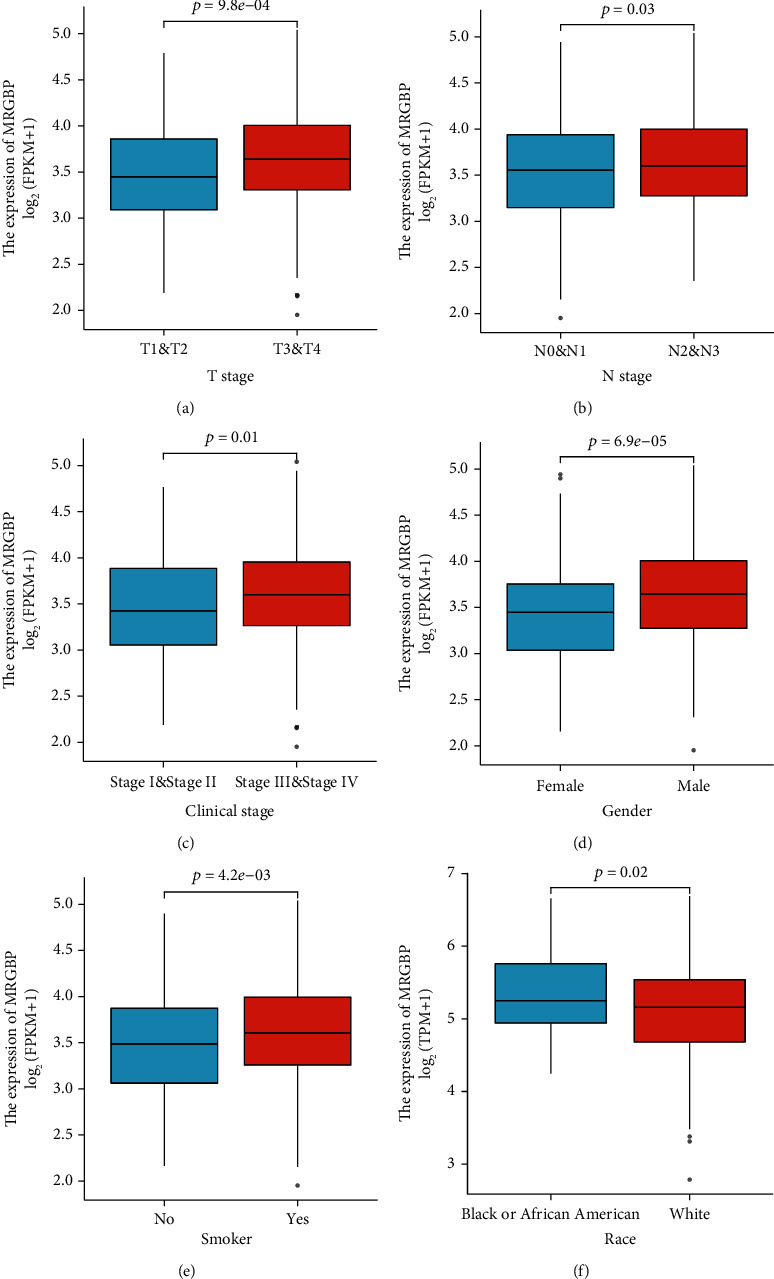
Box plot assessing MRGBP expression of patients with HNSC according to different clinical characteristics. (a) T classification. (b) N classification. (c) Pathological stage. (d) Gender. (e) Smoker. (f) Race.

Clinical and gene expression profiles of 502 HNSC patients were extracted from TCGA database. HNSC patients were divided into high expression group and low expression group according to the median MRGBP expression level (Table 1). The Wilcoxon sign rank and logistic regression were used to analyze the correlation between MRGBP expression and clinical features. High expression of MRGBP was significantly related to T-type (*P* = 9.8E − 04), N-type (*P* = 0.03), and pathological stage (*P* = 0.01). In addition, the high expression of MRGBP was significantly correlated with gender (*P* = 6.9E − 05), smoking (*P* = 4.2E − 03), and race (*P* = 0.02) (Figure 2). After excluding unqualified cases, univariate logistic regression analysis showed that MRGBP expression in HNSC patients was correlated with the clinical characteristics of poor prognosis (Table 2). High MRGBP expression was significantly correlated with gender (OR = 1.825, 95% CI = 1.222 − 2.745, *P* = 0.004), T classification (T3 and T4 vs. T2 and T1: OR = 1.508, 95% CI = 1.040 − 2.193, *P* = 0.031), and smoker (yes vs. no: OR = 1.666, 95% CI = 1.087 − 2.574, *P* = 0.020).

### 3.3. Independent Risk and Diagnostic Value of MRGBP Expression in HNSC

**Figure 3:**
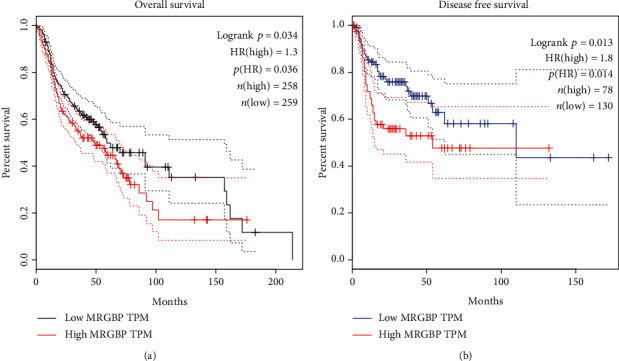
The independent risk and diagnostic value of MRGBP expression in HNSC. The Kaplan-Meier survival analysis of overall survival (OS) (a) and progression-free survival (PFS) (b) in TCGA-HNSC database.

**Figure 4:**
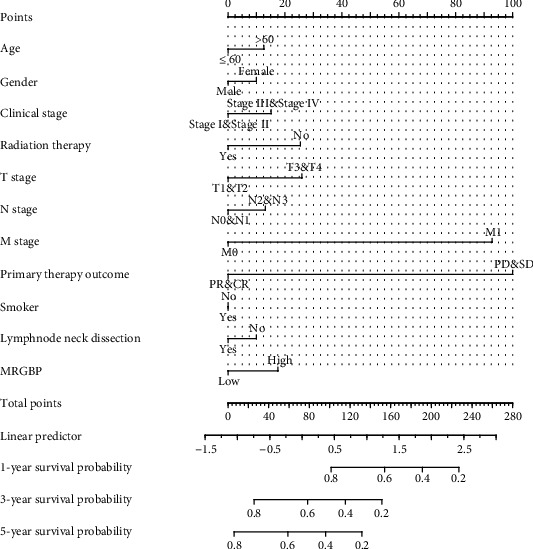
A nomogram for predicting the probability of patients with 1-, 3-, and 5-year overall survival (OS).

**Figure 5:**
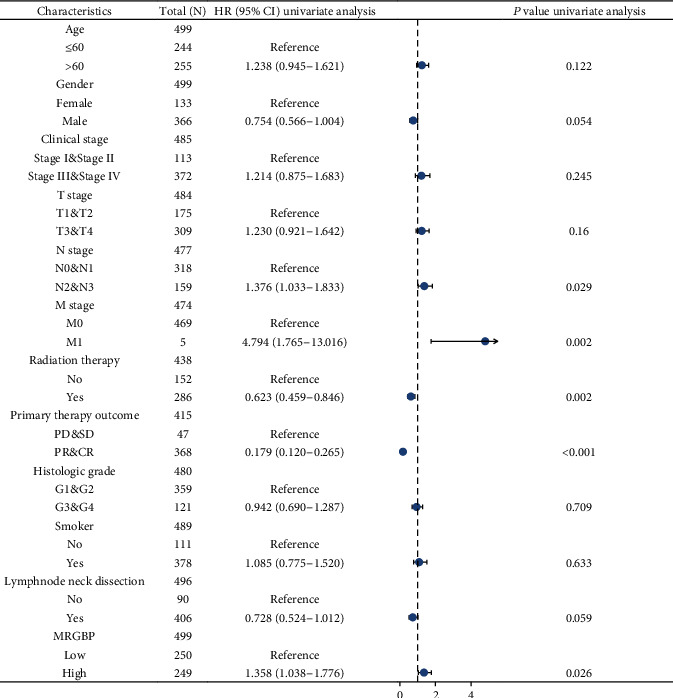
Forest plot of the multivariate Cox regression analysis in TCGA-HNSC.

Survival analysis of the TCGA-HNSC data set showed that high MRGBP expression was significantly correlated with OS (*P* = 0.034) and PFS (*P* = 0.013) (Figure 3). The multivariate Cox analysis showed that high MRGBP expression was significantly associated with OS (hazard ratio (HR) = 1.332, 95% CI = 1.064 − 1.668), disease-specific survival (DSS) (hazard ratio (HR) = 1.460, 95% CI = 1.095 − 1.947), and progression-free interval (PFI) (hazard ratio (HR) = 1.316, 95% CI = 1.040 − 1.666) (Table 3 and Supplementary1, 2). Then, the 1-year, 3-year, and 5-year OS of TCGA-HNSC was predicted according to age, T, M, N grade, pathological stage, and MRGBP (Figure 4). Meanwhile, a forest map was drawn according to CGA-HNSC multifactor Cox regression analysis (Figure 5).

### 3.4. MRGBP-Related Signaling Pathway Based on GSEA

**Figure 6:**
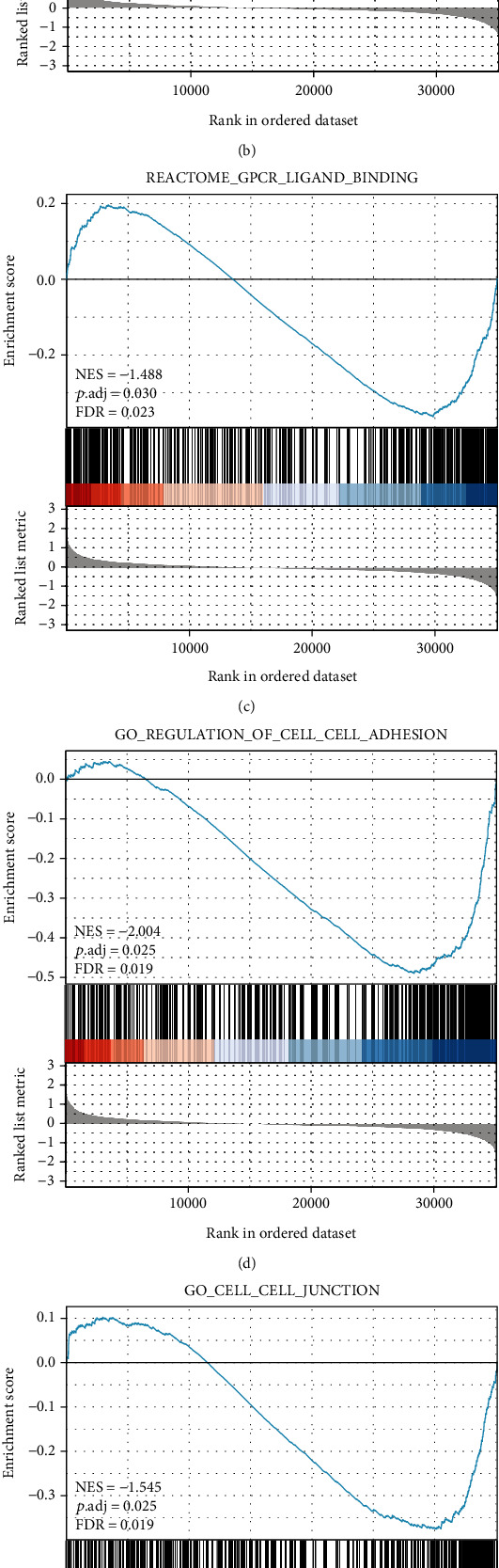
Enrichment plots from GSEA. (a–c) MRGBP-related signaling pathways in c2.cp.v7.2.symbols.gmt. (d–f) MRGBP-related signaling pathways in c5.all.v7.2.symbols.gmt (Gene Ontology).

To explore MRGBP-related signal transduction pathways, we performed gene set enrichment analysis (GSEA). The pathways closely related to the low expression of MRGBP include “neutrophil degranulation,” “signaling by interleukins,” “GPCR ligand binding,” “regulation of cell-cell adhesion,” “cell-cell junction,” and “leukocyte migration” (Figure 6).

### 3.5. Analysis of MRGBP Immune Cell Infiltration in TCGA-HNSC

**Figure 7:**
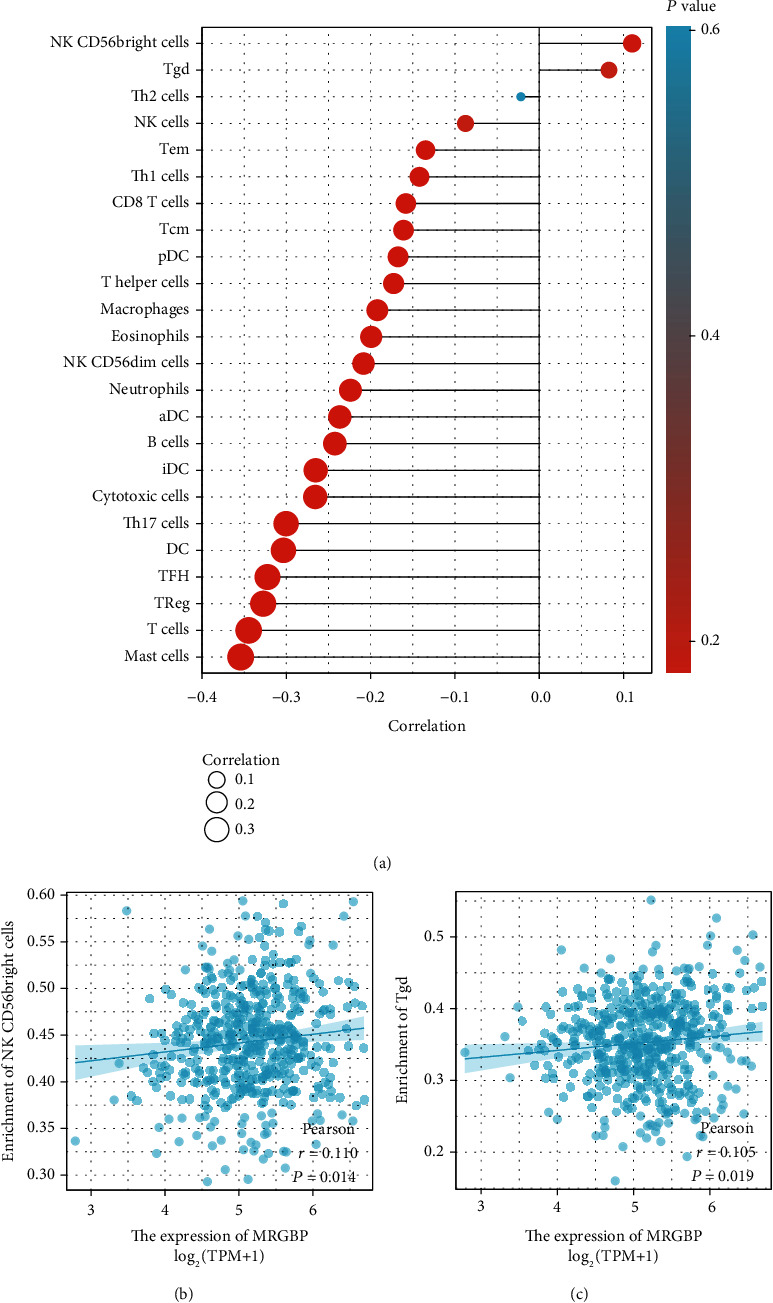
Immune cell infiltration analysis of MRGBP in the TCGA-HNSC. (a) The forest plot shows the correlation between MRGBP expression level and 24 immune cells. The correlation between MRGBP expression and CD56 bright cells (b) and Tgd (c).

The Spearman correlation analysis was used to explore further the relationship between MRGBP and the quantitative resistant cell infiltration level of GSA. The results showed that the high expression of MRGBP was significantly correlated with the infiltration degree of NK CD56 bright cells (*P* = 0.014) and Tgd (*P* = 0.019) (Figure 7).

### 3.6. The Expression of MRGBP

**Figure 8:**
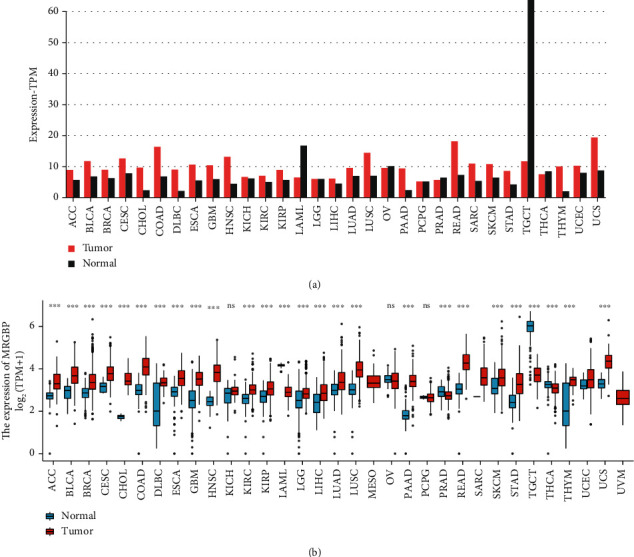
Using GEPIA and TIMER databases to analyze the expression of MRGBP in different tumours. (a) GEPIA database. (b) TIMER database.

The TCGA database further verified the expression of MRGBP, and the results of MRGBP expression in HNSC were consistent with the above results (Figure 8).

## 4. Discussion

MRGBP, identified initially as a novel TRRAP/Tip60 HAT complex component, is encoded by FLJ10914 ORF [5]. The MRG protein family is associated with an eventual loss of cell senescence or proliferation [14]. In addition, the overexpression of MRGBP is involved in the occurrence and development of cancer. MRGBP expression is upregulated in colorectal cancer, and MRGBP plays an essential role in cancer cell proliferation by regulating BRD8 [15]. MRGBP promotes colorectal cancer by playing advantage in cell proliferation or cancer cell division [5]. MRGBP was found to be expressed in human prostate cancer model cells and promote replication and invasion of these cells [16]. MRGBP is frequently upregulated in pancreatic ductal adenocarcinoma tissues and cell lines, and the upregulation of MRGBP is positively correlated with TNM stage and poor prognosis [17]. Of interest, MRGBP promotes the activation of androgen receptor-associated enhancers and promoters through epigenetic mechanisms [18].

Further studies indicated that MRGBP might play an epigenetic regulatory role as a direct downstream target gene of miR-137 in pancreatic cancer [19]. The latest study suggests that MRGBP also plays a role in DNA repair [20]. In this study, we used the TCGA database to study the expression profile of MRGBP in various cancers. The results showed that MRGBP expression was higher in adrenal cortical carcinoma, urothelial bladder carcinoma, hepatocellular carcinoma, oesophagal carcinoma, and head and neck squamous cell carcinoma than in adjacent normal tissues. Chai et al. found that MRGBP is a subunit of the NuA4 histone acetyltransferase complex and participates in the transcriptional activation of particular genes mainly through the acetylation of nucleosome histone H4 and H2A. They explored the expression profile of MRGBP in 33 tumours [21].

In this study, however, we assessed the potential prognostic value of MRGBP in HNSC patients in more detail. Similar to previous studies, in the current study [22], we found that the expression level of MRGBP in HNSC tissues was significantly higher than that in adjacent tissues. The KM plotter analysis showed significant shortening of OS in HNSC patients (*P* < 0.05), and the same was found in the floor of mouth subtype (*P* < 0.05). In addition, the high expression of MRGBP was related to the clinicopathological characteristics of HNSC, including T-type, N-type, pathological stage, and lymph node neck dissection. The results showed that high expression of MRGBP was associated with advanced head and neck squamous cell carcinoma, suggesting that MRGBP may be a marker for early or advanced gastric cancer. More importantly, the ROC analysis also confirmed its diagnostic value. In recent years, HNSC combined with MRGBP expression has not been reported to predict nomograms. Therefore, we constructed prognostic nomograms including age, T, M, N classification, pathological stage, and MRGBP, which doctors can use to improve the accuracy of identifying high-risk patients.

Furthermore, mutations in DNase I hypersensitive sites (DHS) may alter lung cancer susceptibility by regulating the expression of surrounding genes, a process closely related to MRGBP [23]. For hepatocellular carcinoma (HCC), the high expression of MRGBP was significantly correlated with tumour T stage, pathological stage, histological grade, vascular invasion, tumour protein P53 status, and overall survival. MRGBP has high diagnostic accuracy, and the area under the subject operating characteristic curve is 0.980. GSEA revealed the abundance of tumour-related pathways, such as cell cycle and DNA replication pathways, in the highly expressed MRGBP phenotype. GSA showed that MRGBP expression was significantly correlated with 15 kinds of immune cell infiltration. By the Wilcoxon rank-sum test, the concentration scores of T helper cells (Th), T follicular helper cells, CD56 bright natural killer cells, and Th2 cells in the high expression group of MRGBP were significantly increased. Concentration scores of neutrophils, Th17, dendritic cells (DC), *γδ*T, cytotoxic cells, regulatory T cells, plasmacytoid DC, and immature DC were significantly decreased. MRGBP may be a new biomarker to predict the prognosis of liver cancer and a therapeutic target related to immune infiltration [24].

Our study has some limitations—lack of cellular validation as other hard evidence. In addition, due to data from public databases, there may be some bias due to confounding factors.

Although the carcinogenic pathways of different HNSCS are different in molecular level, this study is a preliminary exploration of the carcinogenic molecules of HNSCS in general and lays a foundation for in-depth research on the specific anatomical location and clinical stage. Moreover, the researchers themselves intend to further refine MRGBP to one or more subtypes of HNSC.

In addition, it is a comparative study of the same individual to use paracancer tissue as the control. Due to the different gene expression levels among different individuals, paracancer tissue as the control can be more convenient to explain the experimental results.

## 5. Conclusions

In conclusion, our study suggests that in HNSC, overexpression of MRGBP is associated with poor prognosis and is considered an independent factor in HNSC patients. In addition, GPCR ligand binding, interleukin receptor binding, and neutrophil degranulation may be regulated by MRGBP, and high MRGBP is associated with T cells and mast cells. In head and neck squamous cell carcinoma, increased expression of MRGBP may be an independent prognostic biomarker and is related to the immune invasion.

## Figures and Tables

**Table 1:** Correlation between MRGBP expression and clinicopathological characteristics of patients with HNSC.

Characteristic	Low expression of MRGBP	High expression of MRGBP	*P*
*n*	251	251	
Age, *n* (%)			0.502
≤60	118 (23.6%)	127 (25.3%)	
>60	132 (26.3%)	124 (24.8%)	
Gender, *n* (%)			0.003
Female	82 (16.3%)	52 (10.4%)	
Male	169 (33.7%)	199 (39.6%)	
Race, *n* (%)			0.310
Asian	6 (1.2%)	4 (0.8%)	
Black or African American	19 (3.9%)	28 (5.8%)	
White	218 (44.9%)	210 (43.3%)	
Smoker, *n* (%)			0.026
No	67 (13.6%)	44 (8.9%)	
Yes	182 (37%)	199 (40.4%)	
Clinical stage, *n* (%)			0.569
Stage I	12 (2.5%)	7 (1.4%)	
Stage II	51 (10.5%)	44 (9%)	
Stage III	49 (10%)	53 (10.9%)	
Stage IV	134 (27.5%)	138 (28.3%)	
T stage, *n* (%)			0.069
T1	19 (3.9%)	14 (2.9%)	
T2	82 (16.8%)	62 (12.7%)	
T3	67 (13.8%)	64 (13.1%)	
T4	77 (15.8%)	102 (20.9%)	
N stage, *n* (%)			0.124
N0	127 (26.5%)	112 (23.3%)	
N1	35 (7.3%)	45 (9.4%)	
N2	77 (16%)	77 (16%)	
N3	1 (0.2%)	6 (1.2%)	
M stage, *n* (%)			0.373
M0	237 (49.7%)	235 (49.3%)	
M1	4 (0.8%)	1 (0.2%)	
Age, median (IQR)	61 (54, 69)	60 (52.5, 67)	0.174

**Table 2:** MRGBP expression associated with clinicopathological characteristics (logistic regression).

Characteristics	Total (*N*)	Odds ratio (OR)	*P* value
Age (>60 vs. ≤60)	499	0.858 (0.604-1.220)	0.394
Gender (male vs. female)	500	1.825 (1.222-2.745)	0.004
Clinical stage (stage III and stage IV vs. stage I and stage II)	486	1.290 (0.847-1.971)	0.237
T stage (T3 and T4 vs. T1 and T2)	485	1.508 (1.040-2.193)	0.031
N stage (N2 and N3 vs. N0 and N1)	478	1.099 (0.751-1.609)	0.627
M stage (M1 vs. M0)	475	0.252 (0.013-1.720)	0.219
Radiation therapy (yes vs. no)	439	0.962 (0.649-1.424)	0.845
Primary therapy outcome (PR and CR vs. PD and SD)	416	0.745 (0.401-1.368)	0.343
Histologic grade (G3 and G4 vs. G1 and G2)	481	1.387 (0.918-2.105)	0.122
Smoker (yes vs. no)	490	1.666 (1.087-2.574)	0.020

**Table 3:** Associations with clinicopathological characteristics in HNSC patients using Cox regression.

Characteristics	Total (*N*)	Univariate analysis		Multivariate analysis	
		Hazard ratio (95% CI)	*P* value	Hazard ratio (95% CI)	*P* value
Age	501				
≤60	245	Reference			
>60	256	1.252 (0.956-1.639)	0.102		
Race	485				
Asian and Black or African American	57	Reference			
White	428	0.680 (0.450-1.028)	0.067	0.843 (0.537-1.322)	0.456
Smoker	491				
No	111	Reference			
Yes	380	1.089 (0.778-1.525)	0.618		
Clinical stage	487				
Stage I and stage II	113	Reference			
Stage III and stage IV	374	1.217 (0.878-1.688)	0.238		
T stage	486				
T1 and T2	176	Reference			
T3 and T4	310	1.245 (0.932-1.661)	0.137		
N stage	479				
N0 and N1	318	Reference			
N2 and N3	161	1.384 (1.040-1.842)	0.026	1.308 (0.972-1.759)	0.076
M stage	476				
M0	471	Reference			
M1	5	4.745 (1.748-12.883)	0.002	4.014 (1.411-11.419)	0.009
MRGBP	501	1.301 (1.052-1.608)	0.015	1.332 (1.064-1.668)	0.012

## Data Availability

The (Figures 5 and 6) data used to support the findings of this study are available from the corresponding author upon request.

## Supplementary Materials

Supplementary Materials Supplementary Materials In addition, multivariate Cox analysis showed that high MRGBP expression was also associated with disease-specific survival (DSS) (hazard ratio (HR) = 1.460, 95% CI = 1.095 − 1.947) and progression-free interval (PFI) (hazard ratio (HR) = 1.316, 95% CI = 1.040 − 1.666). Supplementary 1: associations with clinicopathological characteristics for DSS in HNSC patients using the Cox regression. Supplementary 2: associations with clinicopathological characteristics for PFI in HNSC patients using the Cox regression.
