# Supplementary material for: MRGBP: A New Factor for Diagnosis and Prediction of Head and Neck Squamous Cell Carcinoma
Source: Biomed Res Int. 2022 Jul 25;2022:7281120. doi: 10.1155/2022/7281120 (PMC9343194; doi:10.1155/2022/7281120)
Supplement: Supplementary Materials — In addition, multivariate Cox analysis showed that high MRGBP expression was also associated with disease-specific survival (DSS) (hazard ratio (HR) = 1.460, 95% CI = 1.095 − 1.947) and progression-free interval (PFI) (hazard ratio (HR) = 1.316, 95% CI = 1.040 − 1.666). Supplementary 1: associations with clinicopathological characteristics for DSS in HNSC patients using the Cox regression. Supplementary 2: associations with clinicopathological characteristics for PFI in HNSC patients using the Cox regression. [file 7281120.f1.zip › Supplementary1.docx]

| Characteristics | Total(N) | Univariate analysis | |  | Multivariate analysis | |
| --- | --- | --- | --- | --- | --- | --- |
|  |  | Hazard ratio (95% CI) | P value |  | Hazard ratio (95% CI) | P value |
| Age | 476 |  |  |  |  |  |
| <=60 | 235 | Reference |  |  |  |  |
| >60 | 241 | 1.078 (0.763-1.524) | 0.670 |  |  |  |
| Race | 460 |  |  |  |  |  |
| Asian&Black or African American | 56 | Reference |  |  |  |  |
| White | 404 | 0.698 (0.412-1.181) | 0.180 |  |  |  |
| Smoker | 468 |  |  |  |  |  |
| No | 107 | Reference |  |  |  |  |
| Yes | 361 | 1.034 (0.679-1.574) | 0.877 |  |  |  |
| Clinical stage | 462 |  |  |  |  |  |
| Stage I&Stage II | 106 | Reference |  |  |  |  |
| Stage III&Stage IV | 356 | 1.151 (0.753-1.760) | 0.517 |  |  |  |
| T stage | 461 |  |  |  |  |  |
| T1&T2 | 166 | Reference |  |  |  |  |
| T3&T4 | 295 | 1.459 (0.988-2.153) | 0.057 |  | 1.416 (0.935-2.142) | 0.100 |
| N stage | 454 |  |  |  |  |  |
| N0&N1 | 297 | Reference |  |  |  |  |
| N2&N3 | 157 | 1.655 (1.159-2.362) | **0.006** |  | 1.607 (1.119-2.308) | **0.010** |
| M stage | 451 |  |  |  |  |  |
| M0 | 447 | Reference |  |  |  |  |
| M1 | 4 | 8.056 (2.527-25.680) | **<0.001** |  | 10.381 (3.163-34.070) | **<0.001** |
| MRGBP | 476 | 1.479 (1.122-1.949) | **0.005** |  | 1.460 (1.095-1.947) | **0.010** |
