# Supplementary material for: MRGBP: A New Factor for Diagnosis and Prediction of Head and Neck Squamous Cell Carcinoma
Source: Biomed Res Int. 2022 Jul 25;2022:7281120. doi: 10.1155/2022/7281120 (PMC9343194; doi:10.1155/2022/7281120)
Supplement: Supplementary Materials — In addition, multivariate Cox analysis showed that high MRGBP expression was also associated with disease-specific survival (DSS) (hazard ratio (HR) = 1.460, 95% CI = 1.095 − 1.947) and progression-free interval (PFI) (hazard ratio (HR) = 1.316, 95% CI = 1.040 − 1.666). Supplementary 1: associations with clinicopathological characteristics for DSS in HNSC patients using the Cox regression. Supplementary 2: associations with clinicopathological characteristics for PFI in HNSC patients using the Cox regression. [file 7281120.f1.zip › Supplementary2.docx]

| Characteristics | Total(N) | Univariate analysis | |  | Multivariate analysis | |
| --- | --- | --- | --- | --- | --- | --- |
|  |  | Hazard ratio (95% CI) | P value |  | Hazard ratio (95% CI) | P value |
| Age | 501 |  |  |  |  |  |
| <=60 | 245 | Reference |  |  |  |  |
| >60 | 256 | 1.078 (0.812-1.431) | 0.604 |  |  |  |
| Race | 485 |  |  |  |  |  |
| Asian&Black or African American | 57 | Reference |  |  |  |  |
| White | 428 | 0.709 (0.461-1.091) | 0.118 |  |  |  |
| Smoker | 491 |  |  |  |  |  |
| No | 111 | Reference |  |  |  |  |
| Yes | 380 | 0.892 (0.641-1.240) | 0.496 |  |  |  |
| Clinical stage | 487 |  |  |  |  |  |
| Stage I&Stage II | 113 | Reference |  |  |  |  |
| Stage III&Stage IV | 374 | 1.192 (0.840-1.693) | 0.325 |  |  |  |
| T stage | 486 |  |  |  |  |  |
| T1&T2 | 176 | Reference |  |  |  |  |
| T3&T4 | 310 | 1.342 (0.982-1.833) | 0.065 |  | 1.218 (0.881-1.683) | 0.233 |
| N stage | 479 |  |  |  |  |  |
| N0&N1 | 318 | Reference |  |  |  |  |
| N2&N3 | 161 | 1.446 (1.070-1.954) | **0.016** |  | 1.395 (1.025-1.897) | **0.034** |
| M stage | 476 |  |  |  |  |  |
| M0 | 471 | Reference |  |  |  |  |
| M1 | 5 | 2.827 (0.900-8.885) | 0.075 |  | 2.823 (0.892-8.935) | 0.077 |
| MRGBP | 501 | 1.364 (1.089-1.709) | **0.007** |  | 1.316 (1.040-1.666) | **0.022** |
